# (Acetyl­acetonato-κ^2^ *O*,*O*′)carbon­yl{dicyclo­hex­yl[4-(dimethyl­amino)­phen­yl]phosphane-κ*P*}rhodium(I)

**DOI:** 10.1107/S1600536811050483

**Published:** 2011-11-30

**Authors:** Wade L. Davis, Reinout Meijboom

**Affiliations:** a Research Center for Synthesis and Catalysis, Department of Chemistry, University of Johannesburg (APK Campus), PO Box 524, Auckland Park, Johannesburg, 2006, South Africa

## Abstract

The title compound, [Rh(C_5_H_7_O_2_)(C_20_H_32_NP)(CO)], features an acetyl­acetonate-chelated Rh^I^ cation coordinated by one P [Rh—P = 2.2525 (7) Å], one carbonyl C [Rh—C = 1.792 (3) Å] and two O [Rh—O = 2.0582 (17) and 2.0912 (18) Å] atoms in a slightly distorted square-planar geometry. Mol­ecules are packed in positions of least steric hindrance, with the phosphane ligands positioned above and below the Rh–acetyl­acetonate backbone.

## Related literature

For background to the catalytic activity of rhodium–phosphane compounds, see: Carraz *et al.* (2000 ▶); Moloy & Wegman (1989 ▶); Bonati & Wilkinson (1964 ▶). For related rhodium compounds, see: Brink *et al.* (2007 ▶).
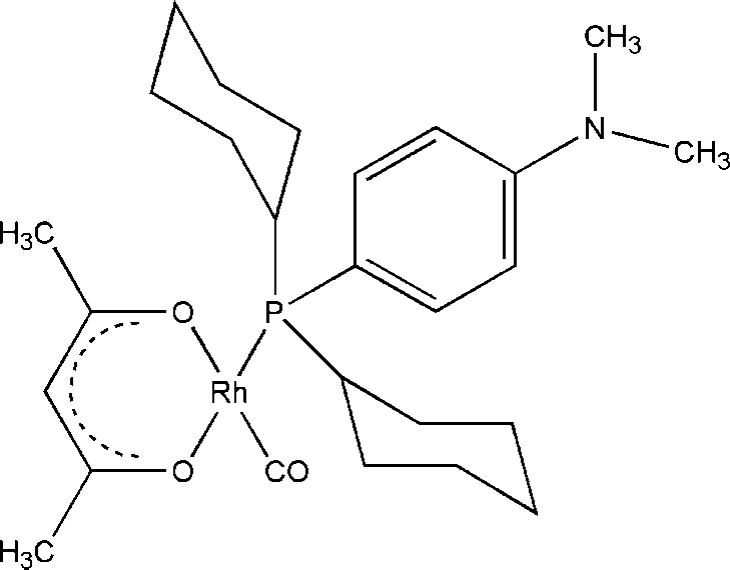


## Experimental

### 

#### Crystal data

[Rh(C_5_H_7_O_2_)(C_20_H_32_NP)(CO)]
*M* *_r_* = 547.46
Monoclinic, 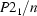

*a* = 12.6865 (9) Å
*b* = 14.5220 (11) Å
*c* = 14.025 (1) Å
β = 93.241 (4)°
*V* = 2579.7 (3) Å^3^
*Z* = 4
Cu *K*α radiation
μ = 6.14 mm^−1^
*T* = 100 K
0.17 × 0.07 × 0.04 mm

#### Data collection

Bruker APEX DUO 4K-CCD diffractometer
Absorption correction: multi-scan (*SADABS*; Bruker, 2008 ▶) *T*_min_ = 0.422, *T*_max_ = 0.791
40437 measured reflections
4303 independent reflections
3693 reflections with *I* > 2σ(*I*)
*R*_int_ = 0.061

#### Refinement

*R*[*F* ^2^ > 2σ(*F* ^2^)] = 0.029
*wR*(*F* ^2^) = 0.076
*S* = 1.12
4303 reflections
293 parameters
H-atom parameters constrained
Δρ_max_ = 0.49 e Å^−3^
Δρ_min_ = −0.71 e Å^−3^

### 

Data collection: *APEX2* (Bruker, 2010 ▶); cell refinement: *SAINT* (Bruker, 2008 ▶); data reduction: *SAINT* and *XPREP* (Bruker, 2008 ▶); program(s) used to solve structure: *SIR97* (Altomare *et al.*, 1999 ▶); program(s) used to refine structure: *SHELXL97* (Sheldrick, 2008 ▶); molecular graphics: *DIAMOND* (Brandenburg & Putz, 2005 ▶); software used to prepare material for publication: *publCIF* (Westrip, 2010 ▶) and *WinGX* (Farrugia, 1999 ▶).

## Supplementary Material

Crystal structure: contains datablock(s) global, I. DOI: 10.1107/S1600536811050483/mw2037sup1.cif

Structure factors: contains datablock(s) I. DOI: 10.1107/S1600536811050483/mw2037Isup2.hkl

Additional supplementary materials:  crystallographic information; 3D view; checkCIF report

## Figures and Tables

**Fig. 1.**
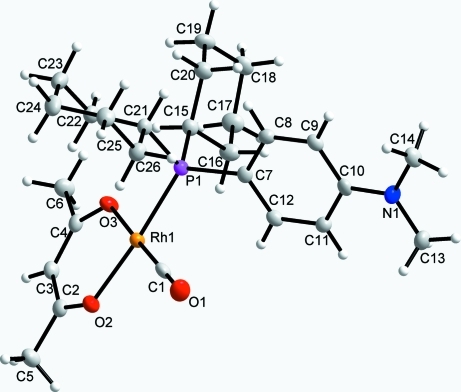
Molecular structure of the title compound, showing the atom numbering system. Displacement ellipsoids are drawn at the 50% probability level. Hydrogen atom labels have been omitted for clarity.

## supplementary crystallographic information

### Comment

Acetylacetonate has two O-donor atoms with equivalent σ-electron donor capabilities. The high symmetry of dicarbonyl(acetylacetonate)rhodium(I) complexes promotes easy carbonyl displacement of either carbonyl group with a variety of phosphanes, phosphites and arsines. (Bonati and Wilkinson, 1964). This work is part of an ongoing investigation aimed at determing the steric effects induced by various phosphane ligands on a rhodium(I) metal centre. Previous work illustrating the catalytic importance of the rhodium(I) square-planar moieties has been conducted on rhodium mono- and di-phosphane complexes containing the symmetrical bidentate ligand, acac (acac = acetylacetonate) (Moloy and Wegman, 1989). Symmetrical di-phosphane ligands result in the production of acetaldehyde, whereas unsymmetrical di-phosphane ligands are more stable and efficient catalysts for the carbonylation of methanol to acetic acid (Carraz *et al.*, 2000).

In the title compound, [Rh(acac)(CO){PCy_2_(4-Me_2_NC_6_H_4_)}] (acac = acetylacetonate, Cy = cyclohexyl), the coordination around the Rh atom shows a slightly distorted square-planar arrangement, illustrated by C1—Rh1—P1 and O2—Rh1—O3 angles of 89.59 (9)° and 88.76 (7)°, respectively. The complex crystallizes in the monoclinic space group, P2(1)/n, with four molecules in the unit cell. A larger *trans* influence of the phosphane ligand with respect to the carbonyl ligand is indicated by the longer Rh—O2 (2.0912 (18) Å) bond compared to Rh—O3 (2.0582 (17) Å) bond which is *trans* to the carbonyl ligand. The steric demand of the phosphane is indicated by the smaller O3—Rh1—P1 angle, (89.36 (5)°), compared to the carbonyl ligand (O2—Rh1—C1= 92.36 (10)°).

Spectroscopic characteristics of the current compound are similar to that reported previously by Brink *et al.* (2007), and we refer the reader to Brink *et al.* (2007) for additional discussion on the spectroscopy of these types of compounds.

### Experimental

A solution of [Rh(acac)(CO)_2_] (25.8 mg, 0.1 mmol) in acetone (5 cm^3^) was slowly added to a solution of [PCy_2_(4-Me_2_NC_6_H_4_)] (31.7 mg, 0.1 mmol) in acetone (5 cm^3^). Slow evaporation of the solvent afforded the title compound as yellow crystals.

### Refinement

The aromatic, methine, and methyl H atoms were placed in geometrically idealized positions (C—H = 0.95–0.98) and constrained to ride on their parent atoms, with *U*_iso_(H) = 1.2*U*_eq_(C) for aromatic and methine H atoms, and *U*_iso_(H) = 1.5*U*_eq_(C) for methyl H atoms respectively. Methyl torsion angles were refined from electron density.

One of the collected sub-sets contained non-reliable data at higher θ angles. In order to obtain reliable data the maximum angle (θ_max_) was cut to 65.03° using the *OMIT* command during refinement cycles.

### Crystal data

**Table:**

[Rh(C_5_H_7_O_2_)(C_20_H_32_NP)(CO)]	*F*(000) = 1144
*M**_r_* = 547.46	*D*_x_ = 1.410 Mg m^−^^3^
Monoclinic, *P*2_1_/*n*	Cu *K*α radiation, λ = 1.54178 Å
Hall symbol: -P 2yn	Cell parameters from 9888 reflections
*a* = 12.6865 (9) Å	θ = 4.4–66.0°
*b* = 14.5220 (11) Å	µ = 6.14 mm^−^^1^
*c* = 14.025 (1) Å	*T* = 100 K
β = 93.241 (4)°	Triangular, yellow
*V* = 2579.7 (3) Å^3^	0.17 × 0.07 × 0.04 mm
*Z* = 4	

### Data collection

**Table:**

Bruker APEX DUO 4K-CCD diffractometer	4303 independent reflections
Radiation source: Incoatec IµS microfocus X-ray source	3693 reflections with *I* > 2σ(*I*)
Incoatec Quazar Multilayer Mirror	*R*_int_ = 0.061
Detector resolution: 8.4 pixels mm^-1^	θ_max_ = 65.0°, θ_min_ = 4.4°
φ and ω scans	*h* = −14→13
Absorption correction: multi-scan (*SADABS*; Bruker, 2008)	*k* = −16→16
*T*_min_ = 0.422, *T*_max_ = 0.791	*l* = −13→16
40437 measured reflections	

### Refinement

**Table:**

Refinement on *F*^2^	Primary atom site location: structure-invariant direct methods
Least-squares matrix: full	Secondary atom site location: difference Fourier map
*R*[*F*^2^ > 2σ(*F*^2^)] = 0.029	Hydrogen site location: inferred from neighbouring sites
*wR*(*F*^2^) = 0.076	H-atom parameters constrained
*S* = 1.12	*w* = 1/[σ^2^(*F*_o_^2^) + (0.0408*P*)^2^ + 0.630*P*] where *P* = (*F*_o_^2^ + 2*F*_c_^2^)/3
4303 reflections	(Δ/σ)_max_ = 0.002
293 parameters	Δρ_max_ = 0.49 e Å^−^^3^
0 restraints	Δρ_min_ = −0.71 e Å^−^^3^

### Special details

**Table:**

Experimental. The intensity data was collected on a Bruker Apex DUO 4 K CCD diffractometer using an exposure time of 10 s/frame. A total of 4784 frames were collected with a frame width of 1.5° covering up to θ = 65.03° with 97.9% completeness accomplished.
Geometry. All e.s.d.'s (except the e.s.d. in the dihedral angle between two l.s. planes) are estimated using the full covariance matrix. The cell e.s.d.'s are taken into account individually in the estimation of e.s.d.'s in distances, angles and torsion angles; correlations between e.s.d.'s in cell parameters are only used when they are defined by crystal symmetry. An approximate (isotropic) treatment of cell e.s.d.'s is used for estimating e.s.d.'s involving l.s. planes.
Refinement. Refinement of *F*^2^ against ALL reflections. The weighted *R*-factor *wR* and goodness of fit *S* are based on *F*^2^, conventional *R*-factors *R* are based on *F*, with *F* set to zero for negative *F*^2^. The threshold expression of *F*^2^ > σ(*F*^2^) is used only for calculating *R*-factors(gt) *etc*. and is not relevant to the choice of reflections for refinement. *R*-factors based on *F*^2^ are statistically about twice as large as those based on *F*, and *R*- factors based on ALL data will be even larger.

### Fractional atomic coordinates and isotropic or equivalent isotropic displacement parameters (Å^2^)

**Table:**

	*x*	*y*	*z*	*U*_iso_*/*U*_eq_	
Rh1	0.908529 (14)	0.744727 (13)	0.325453 (13)	0.01657 (9)	
P1	0.83169 (5)	0.87425 (5)	0.26682 (5)	0.01694 (16)	
O1	0.72624 (15)	0.71456 (15)	0.44366 (14)	0.0305 (5)	
O3	1.03538 (14)	0.76915 (13)	0.24378 (14)	0.0227 (4)	
O2	0.97876 (13)	0.62028 (12)	0.36911 (13)	0.0208 (4)	
N1	0.58506 (16)	1.10132 (17)	0.53812 (16)	0.0240 (5)	
C1	0.7979 (2)	0.72634 (19)	0.3971 (2)	0.0220 (6)	
C7	0.75721 (18)	0.94435 (18)	0.34654 (18)	0.0174 (6)	
C8	0.67428 (18)	1.00226 (18)	0.31360 (19)	0.0186 (6)	
H3	0.6565	1.0058	0.2470	0.022*	
C9	0.61789 (18)	1.05425 (18)	0.37573 (18)	0.0187 (6)	
H4	0.5618	1.0922	0.3511	0.022*	
C10	0.64221 (18)	1.05186 (18)	0.47480 (18)	0.0175 (6)	
C13	0.6132 (2)	1.0988 (2)	0.63920 (19)	0.0277 (7)	
H6A	0.6049	1.0359	0.6630	0.042*	
H6B	0.5670	1.1404	0.6727	0.042*	
H6C	0.6868	1.1183	0.6506	0.042*	
C14	0.49332 (19)	1.1551 (2)	0.5047 (2)	0.0245 (6)	
H7A	0.5166	1.2102	0.4714	0.037*	
H7B	0.4534	1.1736	0.5595	0.037*	
H7C	0.4482	1.1178	0.4609	0.037*	
C11	0.72802 (18)	0.99636 (18)	0.50780 (19)	0.0198 (6)	
H8	0.7486	0.9951	0.5740	0.024*	
C12	0.78251 (18)	0.94380 (18)	0.44461 (18)	0.0189 (6)	
H9	0.8390	0.9061	0.4689	0.023*	
C15	0.93139 (18)	0.95227 (19)	0.21934 (18)	0.0194 (6)	
H10	0.9798	0.9127	0.1832	0.023*	
C16	0.99933 (19)	0.9952 (2)	0.3020 (2)	0.0247 (6)	
H11A	0.9550	1.0362	0.3395	0.030*	
H11B	1.0278	0.9459	0.3449	0.030*	
C17	1.0909 (2)	1.0506 (2)	0.2639 (2)	0.0281 (7)	
H12A	1.1300	1.0816	0.3179	0.034*	
H12B	1.1401	1.0080	0.2339	0.034*	
C18	1.0528 (2)	1.12267 (19)	0.1909 (2)	0.0253 (6)	
H13A	1.0122	1.1707	0.2230	0.030*	
H13B	1.1146	1.1526	0.1638	0.030*	
C19	0.9839 (2)	1.0799 (2)	0.1111 (2)	0.0252 (6)	
H14A	1.0269	1.0371	0.0743	0.030*	
H14B	0.9568	1.1289	0.0671	0.030*	
C20	0.8904 (2)	1.02713 (19)	0.1499 (2)	0.0233 (6)	
H15A	0.8446	1.0703	0.1833	0.028*	
H15B	0.8479	0.9987	0.0964	0.028*	
C21	0.73975 (18)	0.84942 (19)	0.16375 (18)	0.0189 (6)	
H16	0.7136	0.9092	0.1362	0.023*	
C26	0.64365 (19)	0.7920 (2)	0.19092 (19)	0.0232 (6)	
H17A	0.6683	0.7336	0.2211	0.028*	
H17B	0.6036	0.8264	0.2379	0.028*	
C25	0.5715 (2)	0.7708 (2)	0.1027 (2)	0.0265 (7)	
H18A	0.5431	0.8290	0.0751	0.032*	
H18B	0.5112	0.7330	0.1216	0.032*	
C24	0.6310 (2)	0.7191 (2)	0.0275 (2)	0.0297 (7)	
H19A	0.5835	0.7085	−0.0299	0.036*	
H19B	0.6545	0.6585	0.0530	0.036*	
C23	0.7269 (2)	0.7754 (2)	0.0004 (2)	0.0292 (7)	
H20A	0.7667	0.7402	−0.0461	0.035*	
H20B	0.7025	0.8334	−0.0307	0.035*	
C22	0.7991 (2)	0.7978 (2)	0.08685 (19)	0.0239 (6)	
H21A	0.8584	0.8364	0.0670	0.029*	
H21B	0.8290	0.7400	0.1143	0.029*	
C4	1.11553 (19)	0.7161 (2)	0.23583 (19)	0.0194 (6)	
C6	1.1979 (2)	0.75500 (19)	0.1732 (2)	0.0259 (7)	
H23A	1.1729	0.8137	0.1458	0.039*	
H23B	1.2103	0.7115	0.1216	0.039*	
H23C	1.2639	0.7651	0.2115	0.039*	
C3	1.13142 (18)	0.63104 (18)	0.27803 (18)	0.0203 (6)	
H24	1.1929	0.5983	0.2624	0.024*	
C2	1.06540 (19)	0.58785 (19)	0.34208 (19)	0.0205 (6)	
C5	1.0972 (2)	0.4964 (2)	0.3834 (2)	0.0277 (7)	
H26A	1.0910	0.4977	0.4528	0.042*	
H26B	1.1704	0.4832	0.3693	0.042*	
H26C	1.0509	0.4484	0.3553	0.042*	

### Atomic displacement parameters (Å^2^)

**Table:**

	*U*^11^	*U*^22^	*U*^33^	*U*^12^	*U*^13^	*U*^23^
Rh1	0.01554 (13)	0.01623 (14)	0.01817 (13)	0.00170 (7)	0.00311 (9)	0.00093 (8)
P1	0.0158 (3)	0.0166 (4)	0.0187 (3)	0.0017 (2)	0.0031 (3)	0.0004 (3)
O1	0.0295 (10)	0.0310 (12)	0.0326 (12)	−0.0047 (9)	0.0158 (9)	0.0000 (10)
O3	0.0207 (9)	0.0207 (11)	0.0270 (11)	0.0051 (8)	0.0053 (8)	0.0060 (8)
O2	0.0189 (8)	0.0190 (11)	0.0246 (10)	0.0023 (7)	0.0023 (8)	0.0007 (8)
N1	0.0247 (11)	0.0267 (14)	0.0210 (12)	0.0064 (10)	0.0035 (10)	−0.0028 (10)
C1	0.0272 (15)	0.0127 (15)	0.0257 (16)	0.0040 (11)	−0.0002 (13)	0.0013 (12)
C7	0.0146 (11)	0.0171 (15)	0.0209 (14)	−0.0023 (10)	0.0032 (10)	−0.0012 (11)
C8	0.0186 (12)	0.0180 (15)	0.0191 (14)	−0.0015 (10)	0.0008 (11)	0.0022 (11)
C9	0.0156 (11)	0.0153 (15)	0.0253 (15)	0.0000 (10)	0.0009 (11)	0.0012 (12)
C10	0.0167 (11)	0.0121 (14)	0.0241 (15)	−0.0030 (10)	0.0040 (11)	−0.0011 (11)
C13	0.0349 (15)	0.0265 (17)	0.0224 (15)	0.0025 (13)	0.0079 (13)	−0.0031 (13)
C14	0.0208 (12)	0.0219 (16)	0.0318 (16)	0.0029 (11)	0.0097 (12)	0.0000 (13)
C11	0.0212 (12)	0.0200 (16)	0.0181 (14)	0.0003 (11)	−0.0006 (11)	−0.0004 (11)
C12	0.0148 (11)	0.0172 (15)	0.0246 (15)	0.0006 (10)	0.0007 (10)	0.0018 (12)
C15	0.0178 (12)	0.0187 (15)	0.0218 (14)	0.0005 (10)	0.0029 (11)	−0.0001 (12)
C16	0.0205 (12)	0.0262 (17)	0.0272 (15)	−0.0033 (11)	−0.0014 (11)	0.0046 (13)
C17	0.0195 (13)	0.0297 (18)	0.0350 (17)	−0.0052 (12)	0.0002 (12)	0.0036 (14)
C18	0.0216 (13)	0.0213 (16)	0.0336 (16)	−0.0035 (11)	0.0069 (12)	0.0039 (13)
C19	0.0256 (13)	0.0238 (16)	0.0269 (15)	0.0011 (12)	0.0061 (12)	0.0088 (13)
C20	0.0221 (13)	0.0215 (16)	0.0265 (15)	−0.0008 (11)	0.0025 (11)	0.0023 (13)
C21	0.0197 (12)	0.0183 (15)	0.0191 (14)	0.0021 (11)	0.0044 (11)	−0.0009 (11)
C26	0.0217 (13)	0.0257 (17)	0.0221 (15)	−0.0010 (12)	0.0019 (11)	−0.0015 (12)
C25	0.0229 (14)	0.0284 (17)	0.0276 (17)	0.0024 (12)	−0.0034 (12)	−0.0025 (13)
C24	0.0326 (15)	0.0296 (18)	0.0259 (16)	0.0053 (13)	−0.0080 (13)	−0.0085 (14)
C23	0.0376 (16)	0.0313 (18)	0.0189 (15)	0.0111 (14)	0.0014 (13)	−0.0019 (13)
C22	0.0277 (14)	0.0230 (17)	0.0214 (15)	0.0042 (12)	0.0064 (12)	−0.0008 (13)
C4	0.0163 (12)	0.0234 (16)	0.0184 (14)	0.0016 (11)	0.0004 (11)	−0.0054 (12)
C6	0.0206 (14)	0.0278 (18)	0.0301 (17)	0.0018 (11)	0.0083 (13)	0.0044 (13)
C3	0.0161 (11)	0.0217 (16)	0.0230 (14)	0.0036 (11)	0.0016 (11)	−0.0034 (12)
C2	0.0208 (12)	0.0202 (16)	0.0201 (14)	0.0009 (11)	−0.0014 (11)	−0.0043 (12)
C5	0.0298 (14)	0.0216 (17)	0.0324 (17)	0.0053 (12)	0.0073 (13)	0.0030 (13)

### Geometric parameters (Å, °)

**Table:**

Rh1—C1	1.792 (3)	C17—H12B	0.9900
Rh1—O3	2.0582 (17)	C18—C19	1.515 (4)
Rh1—O2	2.0912 (18)	C18—H13A	0.9900
Rh1—P1	2.2525 (7)	C18—H13B	0.9900
P1—C7	1.816 (2)	C19—C20	1.537 (3)
P1—C21	1.841 (3)	C19—H14A	0.9900
P1—C15	1.850 (2)	C19—H14B	0.9900
O1—C1	1.161 (3)	C20—H15A	0.9900
O3—C4	1.285 (3)	C20—H15B	0.9900
O2—C2	1.273 (3)	C21—C26	1.543 (3)
N1—C10	1.379 (3)	C21—C22	1.544 (3)
N1—C13	1.443 (3)	C21—H16	1.0000
N1—C14	1.457 (3)	C26—C25	1.528 (4)
C7—C12	1.395 (4)	C26—H17A	0.9900
C7—C8	1.405 (4)	C26—H17B	0.9900
C8—C9	1.383 (3)	C25—C24	1.528 (4)
C8—H3	0.9500	C25—H18A	0.9900
C9—C10	1.407 (4)	C25—H18B	0.9900
C9—H4	0.9500	C24—C23	1.531 (4)
C10—C11	1.411 (4)	C24—H19A	0.9900
C13—H6A	0.9800	C24—H19B	0.9900
C13—H6B	0.9800	C23—C22	1.513 (4)
C13—H6C	0.9800	C23—H20A	0.9900
C14—H7A	0.9800	C23—H20B	0.9900
C14—H7B	0.9800	C22—H21A	0.9900
C14—H7C	0.9800	C22—H21B	0.9900
C11—C12	1.384 (3)	C4—C3	1.380 (4)
C11—H8	0.9500	C4—C6	1.512 (4)
C12—H9	0.9500	C6—H23A	0.9800
C15—C20	1.530 (4)	C6—H23B	0.9800
C15—C16	1.537 (4)	C6—H23C	0.9800
C15—H10	1.0000	C3—C2	1.410 (4)
C16—C17	1.534 (3)	C3—H24	0.9500
C16—H11A	0.9900	C2—C5	1.495 (4)
C16—H11B	0.9900	C5—H26A	0.9800
C17—C18	1.523 (4)	C5—H26B	0.9800
C17—H12A	0.9900	C5—H26C	0.9800
			
C1—Rh1—O3	178.64 (10)	H13A—C18—H13B	108.0
C1—Rh1—O2	92.36 (10)	C18—C19—C20	111.5 (2)
O3—Rh1—O2	88.76 (7)	C18—C19—H14A	109.3
C1—Rh1—P1	89.59 (9)	C20—C19—H14A	109.3
O3—Rh1—P1	89.36 (5)	C18—C19—H14B	109.3
O2—Rh1—P1	175.54 (5)	C20—C19—H14B	109.3
C7—P1—C21	105.34 (11)	H14A—C19—H14B	108.0
C7—P1—C15	105.57 (11)	C15—C20—C19	109.8 (2)
C21—P1—C15	104.66 (12)	C15—C20—H15A	109.7
C7—P1—Rh1	118.21 (9)	C19—C20—H15A	109.7
C21—P1—Rh1	111.40 (9)	C15—C20—H15B	109.7
C15—P1—Rh1	110.63 (9)	C19—C20—H15B	109.7
C4—O3—Rh1	126.32 (17)	H15A—C20—H15B	108.2
C2—O2—Rh1	126.39 (16)	C26—C21—C22	109.5 (2)
C10—N1—C13	120.6 (2)	C26—C21—P1	112.73 (17)
C10—N1—C14	120.8 (2)	C22—C21—P1	109.34 (17)
C13—N1—C14	118.6 (2)	C26—C21—H16	108.4
O1—C1—Rh1	179.9 (3)	C22—C21—H16	108.4
C12—C7—C8	117.0 (2)	P1—C21—H16	108.4
C12—C7—P1	120.35 (19)	C25—C26—C21	110.8 (2)
C8—C7—P1	122.6 (2)	C25—C26—H17A	109.5
C9—C8—C7	121.6 (2)	C21—C26—H17A	109.5
C9—C8—H3	119.2	C25—C26—H17B	109.5
C7—C8—H3	119.2	C21—C26—H17B	109.5
C8—C9—C10	121.1 (2)	H17A—C26—H17B	108.1
C8—C9—H4	119.4	C26—C25—C24	111.2 (2)
C10—C9—H4	119.4	C26—C25—H18A	109.4
N1—C10—C9	121.9 (2)	C24—C25—H18A	109.4
N1—C10—C11	120.7 (2)	C26—C25—H18B	109.4
C9—C10—C11	117.4 (2)	C24—C25—H18B	109.4
N1—C13—H6A	109.5	H18A—C25—H18B	108.0
N1—C13—H6B	109.5	C25—C24—C23	109.9 (3)
H6A—C13—H6B	109.5	C25—C24—H19A	109.7
N1—C13—H6C	109.5	C23—C24—H19A	109.7
H6A—C13—H6C	109.5	C25—C24—H19B	109.7
H6B—C13—H6C	109.5	C23—C24—H19B	109.7
N1—C14—H7A	109.5	H19A—C24—H19B	108.2
N1—C14—H7B	109.5	C22—C23—C24	111.7 (2)
H7A—C14—H7B	109.5	C22—C23—H20A	109.3
N1—C14—H7C	109.5	C24—C23—H20A	109.3
H7A—C14—H7C	109.5	C22—C23—H20B	109.3
H7B—C14—H7C	109.5	C24—C23—H20B	109.3
C12—C11—C10	120.6 (2)	H20A—C23—H20B	107.9
C12—C11—H8	119.7	C23—C22—C21	111.5 (2)
C10—C11—H8	119.7	C23—C22—H21A	109.3
C11—C12—C7	122.2 (2)	C21—C22—H21A	109.3
C11—C12—H9	118.9	C23—C22—H21B	109.3
C7—C12—H9	118.9	C21—C22—H21B	109.3
C20—C15—C16	110.3 (2)	H21A—C22—H21B	108.0
C20—C15—P1	116.72 (17)	O3—C4—C3	126.6 (2)
C16—C15—P1	110.03 (17)	O3—C4—C6	113.8 (2)
C20—C15—H10	106.4	C3—C4—C6	119.6 (2)
C16—C15—H10	106.4	C4—C6—H23A	109.5
P1—C15—H10	106.4	C4—C6—H23B	109.5
C17—C16—C15	110.7 (2)	H23A—C6—H23B	109.5
C17—C16—H11A	109.5	C4—C6—H23C	109.5
C15—C16—H11A	109.5	H23A—C6—H23C	109.5
C17—C16—H11B	109.5	H23B—C6—H23C	109.5
C15—C16—H11B	109.5	C4—C3—C2	126.4 (2)
H11A—C16—H11B	108.1	C4—C3—H24	116.8
C18—C17—C16	112.2 (2)	C2—C3—H24	116.8
C18—C17—H12A	109.2	O2—C2—C3	125.4 (3)
C16—C17—H12A	109.2	O2—C2—C5	115.6 (2)
C18—C17—H12B	109.2	C3—C2—C5	119.0 (2)
C16—C17—H12B	109.2	C2—C5—H26A	109.5
H12A—C17—H12B	107.9	C2—C5—H26B	109.5
C19—C18—C17	111.3 (2)	H26A—C5—H26B	109.5
C19—C18—H13A	109.4	C2—C5—H26C	109.5
C17—C18—H13A	109.4	H26A—C5—H26C	109.5
C19—C18—H13B	109.4	H26B—C5—H26C	109.5
C17—C18—H13B	109.4		
			
C1—Rh1—P1—C7	36.35 (13)	C7—P1—C15—C16	56.9 (2)
O3—Rh1—P1—C7	−142.79 (11)	C21—P1—C15—C16	167.77 (18)
C1—Rh1—P1—C21	−85.84 (12)	Rh1—P1—C15—C16	−72.14 (18)
O3—Rh1—P1—C21	95.02 (9)	C20—C15—C16—C17	−56.8 (3)
C1—Rh1—P1—C15	158.19 (13)	P1—C15—C16—C17	173.02 (18)
O3—Rh1—P1—C15	−20.95 (11)	C15—C16—C17—C18	54.4 (3)
O2—Rh1—O3—C4	0.2 (2)	C16—C17—C18—C19	−53.7 (3)
P1—Rh1—O3—C4	−175.7 (2)	C17—C18—C19—C20	55.4 (3)
C1—Rh1—O2—C2	179.1 (2)	C16—C15—C20—C19	58.4 (3)
O3—Rh1—O2—C2	−1.7 (2)	P1—C15—C20—C19	−175.11 (18)
C21—P1—C7—C12	152.7 (2)	C18—C19—C20—C15	−58.0 (3)
C15—P1—C7—C12	−96.8 (2)	C7—P1—C21—C26	−63.5 (2)
Rh1—P1—C7—C12	27.5 (2)	C15—P1—C21—C26	−174.56 (18)
C21—P1—C7—C8	−28.6 (2)	Rh1—P1—C21—C26	65.87 (19)
C15—P1—C7—C8	81.8 (2)	C7—P1—C21—C22	174.49 (18)
Rh1—P1—C7—C8	−153.80 (18)	C15—P1—C21—C22	63.4 (2)
C12—C7—C8—C9	−2.0 (4)	Rh1—P1—C21—C22	−56.16 (19)
P1—C7—C8—C9	179.28 (19)	C22—C21—C26—C25	−56.4 (3)
C7—C8—C9—C10	0.6 (4)	P1—C21—C26—C25	−178.32 (18)
C13—N1—C10—C9	−178.8 (2)	C21—C26—C25—C24	58.0 (3)
C14—N1—C10—C9	2.9 (4)	C26—C25—C24—C23	−57.0 (3)
C13—N1—C10—C11	1.3 (4)	C25—C24—C23—C22	56.4 (3)
C14—N1—C10—C11	−177.0 (2)	C24—C23—C22—C21	−56.6 (3)
C8—C9—C10—N1	−178.2 (2)	C26—C21—C22—C23	55.9 (3)
C8—C9—C10—C11	1.7 (4)	P1—C21—C22—C23	179.8 (2)
N1—C10—C11—C12	177.3 (2)	Rh1—O3—C4—C3	2.1 (4)
C9—C10—C11—C12	−2.6 (4)	Rh1—O3—C4—C6	−177.80 (17)
C10—C11—C12—C7	1.3 (4)	O3—C4—C3—C2	−3.6 (5)
C8—C7—C12—C11	1.1 (4)	C6—C4—C3—C2	176.3 (3)
P1—C7—C12—C11	179.8 (2)	Rh1—O2—C2—C3	1.0 (4)
C7—P1—C15—C20	−69.8 (2)	Rh1—O2—C2—C5	−179.18 (18)
C21—P1—C15—C20	41.1 (2)	C4—C3—C2—O2	1.9 (5)
Rh1—P1—C15—C20	161.20 (17)	C4—C3—C2—C5	−178.0 (3)
